# Mesenchymal Stem Cell-Derived Extracellular Vesicles in Myocardial Ischemia–Reperfusion Injury: A Comprehensive Review

**DOI:** 10.3390/biology15050383

**Published:** 2026-02-26

**Authors:** Luca Bonanni, Nicola Ferri

**Affiliations:** 1 Department of Medicine, Ospedale Dell’Angelo, 30174 Venice, Italy; 2 Department of Medicine, University of Padua, 35122 Padova, Italy; nicola.ferri@unipd.it

**Keywords:** mesenchymal stem cells, extracellular vesicles, ischemia–reperfusion injury, myocardial infarction, cardioprotection, mitochondrial dysfunction, inflammation, signaling pathways

## Abstract

Myocardial ischemia–reperfusion injury remains a major unresolved challenge in cardiovascular medicine. Although timely restoration of blood flow is essential to limit ischemic damage, reperfusion triggers a complex network of maladaptive biological responses, including oxidative stress, calcium overload, mitochondrial dysfunction, metabolic impairment, and sterile inflammation. These processes converge on cardiomyocyte death, adverse ventricular remodeling, and long-term functional deterioration. Mesenchymal stem cells have been widely investigated as cardioprotective agents; however, accumulating evidence indicates that their beneficial effects are predominantly mediated by paracrine mechanisms. Among these, extracellular vesicles released by mesenchymal stem cells have emerged as key biological effectors. Experimental studies demonstrate that mesenchymal stem cell–derived extracellular vesicles modulate multiple signaling pathways involved in ischemia–reperfusion injury, including activation of the phosphoinositide 3-kinase (PI3K) and protein kinase B (PKB) axis, regulation of signal transducer and activator of transcription 3 (STAT3) signaling in a cell-specific manner, suppression of nuclear factor kappa B (NF-κB)-driven inflammatory responses, and stabilization of hypoxia-inducible factor-1α (HIF-1α)–dependent adaptive programs. At the subcellular level, these vesicles preserve mitochondrial structure and function, support energy metabolism, regulate mitophagy, and limit oxidative damage. Their molecular cargo, comprising regulatory microRNAs, metabolic enzymes, and stress-response proteins, enables coordinated modulation of survival, inflammatory, and reparative pathways rather than single-target effects. This review synthesizes current experimental evidence on the mechanistic basis of mesenchymal stem cell–derived extracellular vesicle–mediated cardioprotection and discusses their potential as cell-free, mechanism-based therapeutic strategies to limit myocardial ischemia–reperfusion injury.

## Simple Summary

Restoring blood flow to the heart after a heart attack is essential to save tissue, but it can also trigger additional injury to heart muscle cells. This paradoxical damage is driven by several biological mechanisms, including excessive oxidative stress, disturbances in calcium balance, failure of energy production, inflammation, and dysfunction of mitochondria, the structures responsible for supplying energy to cells. Current therapies do not specifically target these processes. Mesenchymal stem cells have shown protective effects in experimental models, largely through the release of extracellular vesicles, which are small membrane-bound particles that transfer biological signals between cells. In this review, we describe how extracellular vesicles derived from mesenchymal stem cells influence key molecular pathways involved in heart injury after blood flow restoration. These vesicles deliver regulatory molecules that reduce programmed cell death, preserve mitochondrial function, support cellular energy metabolism, and modulate inflammatory responses in the injured heart. Rather than acting on a single target, extracellular vesicles coordinate multiple protective mechanisms simultaneously. A clearer understanding of these biological actions may support the development of new therapeutic strategies aimed at limiting heart damage after a heart attack and improving long-term cardiac recovery through cell-free, mechanism-based interventions.

## 1. Introduction

Myocardial ischemia–reperfusion (I/R) injury remains a central unresolved problem in cardiovascular biology, representing a paradox in which the restoration of blood flow, essential for tissue survival, becomes a potent driver of additional myocardial damage. Despite substantial advances in reperfusion strategies, including timely revascularization and optimized pharmacological support, the biological sequelae of reperfusion continue to exert a decisive influence on infarct size, ventricular remodeling, arrhythmic vulnerability, and long-term cardiac function [1,2].

At the cellular level, reperfusion injury reflects the abrupt reintroduction of oxygen and metabolic substrates into cardiomyocytes that have adapted to ischemic stress. This sudden shift triggers excessive production of reactive oxygen species, calcium overload, mitochondrial dysfunction, and activation of innate immune pathways. These processes converge on multiple forms of regulated cell death, including apoptosis, necrosis, pyroptosis, and inflammation-driven injury, amplifying myocardial damage beyond the ischemic core [1,2]. Importantly, many of these events occur within minutes to hours after reperfusion, defining a narrow therapeutic window that has proven resistant to conventional pharmacological interventions.

Mesenchymal stem cells (MSCs) have long been explored as a potential regenerative therapy in ischemic heart disease. Early enthusiasm centered on their presumed capacity for engraftment and cardiomyogenic differentiation. However, accumulating experimental and clinical data has progressively reshaped this view. It is now widely accepted that the beneficial effects of MSCs are predominantly mediated by paracrine mechanisms rather than by durable engraftment or direct cardiomyocyte replacement [3,4,5]. MSCs influence cardiomyocyte survival, angiogenesis, inflammation, and tissue remodeling through the release of soluble factors and extracellular vesicles.

Despite encouraging preclinical findings, the clinical translation of MSC-based therapies in acute myocardial I/R has been hampered by intrinsic limitations. These include poor myocardial homing and survival, marked donor- and tissue-dependent heterogeneity, and safety concerns such as microvascular obstruction, ectopic differentiation, and immunogenicity [6,7,8]. Collectively, these issues have driven a conceptual shift away from cell replacement toward the identification of the key paracrine mediators responsible for MSC-induced cardioprotection.

Extracellular vesicles (EVs) released by MSCs have emerged as biologically plausible and therapeutically attractive candidates. A growing body of evidence indicates that most MSC-mediated cardioprotective effects are paracrine in nature and can be largely recapitulated by MSC-derived extracellular vesicles (MSC-EVs). These nanoscale vesicles encapsulate a concentrated cargo of microRNAs, proteins, lipids, and metabolites capable of modulating cardiomyocyte survival, inflammation, angiogenesis, and metabolic adaptation [9,10,11]. Compared with live MSCs, EVs exhibit lower immunogenicity, enhanced stability, and greater potential for standardization, storage, and scalable production—features particularly relevant in acute cardiovascular settings [6,10,12].

From a biological standpoint, MSC-EVs should not be viewed merely as passive by-products of MSC secretion, but rather as dynamic signaling entities capable of orchestrating coordinated adaptive responses across multiple cell types within the injured myocardium. Understanding how MSC-EVs integrate survival, metabolic, and immunological pathways is therefore central to their therapeutic exploitation in myocardial I/R injury.

Although MSC-derived extracellular vesicles have been extensively discussed in the broader context of myocardial infarction and cardiac regeneration, a focused mechanistic synthesis specifically addressing their role during the early ischemia–reperfusion phase remains less clearly defined. Given the abrupt metabolic transitions and mitochondrial stress that characterize reperfusion injury, a more integrated analysis of signaling networks and rapid cargo-mediated mechanisms appears warranted.

In this review, we examine MSC-EV-mediated cardioprotection within an ischemia–reperfusion–centered framework, with particular attention to interconnected survival pathways and mitochondrial quality control processes active during the early reperfusion window.

## 2. Experimental Evidence of MSC-EV—Mediated Cardioprotection in Myocardial I/R

### 2.1. Effects on Infarct Size, Cardiac Function, and Electrical Stability

Extensive preclinical evidence demonstrates that MSC-EVs exert robust and reproducible cardioprotective effects across multiple experimental models of myocardial I/R. In murine and rat systems, intramyocardial, intracoronary, or systemic administration of MSC-EVs consistently reduces infarct size and cardiomyocyte apoptosis compared with vehicle-treated controls. These structural benefits are accompanied by significant improvements in left ventricular systolic function and attenuation of adverse post-ischemic remodeling [13,14,15,16].

Importantly, the beneficial effects of MSC-EVs extend beyond structural preservation. Several studies demonstrate improved myocardial electrical stability during reperfusion, with reduced susceptibility to ventricular arrhythmias. This effect is particularly evident with EVs derived from hypoxia- or anoxia-preconditioned MSCs, suggesting that adaptive changes in EV cargo enhance electrophysiological protection [13]. Comparable cardioprotective effects have been observed using EVs derived from adipose-derived MSCs or pharmacologically primed MSCs, supporting the concept that EV potency is modifiable and responsive to environmental conditioning [14,15,16].

### 2.2. Early Cellular and Molecular Effects

Beyond global functional outcomes, MSC-EVs exert early protective effects at the cellular and molecular levels. Experimental models consistently demonstrate reduced cardiomyocyte apoptosis, enhanced endothelial cell survival, promotion of post-ischemic angiogenesis, and attenuation of inflammatory responses within the reperfused myocardium [13,14,15,16,17,18,19,20]. These early adaptations shape the post-ischemic microenvironment and contribute to sustained myocardial salvage and functional recovery.

Suppression of thioredoxin-interacting protein (TXNIP) and downstream caspase-1 signaling has been identified as a key anti-apoptotic mechanism, preserving cardiomyocyte viability and maintaining expression of survival-associated transcription factors such as GATA4 and Bcl-2 during reperfusion [14,15]. In parallel, hypoxia-enriched MSC-EVs modulate Wnt/β-catenin signaling through microRNA-dependent inhibition of glycogen synthase kinase-3β (GSK-3β), thereby restoring gap-junction integrity and improving post-ischemic electrical conduction [13].

Rather than acting through a single dominant mechanism, MSC-EVs influence multiple interconnected biological processes. This pleiotropic mode of action distinguishes EV-based therapy from conventional single-target pharmacological approaches and likely explains its reproducibility across diverse experimental models of myocardial I/R injury.

## 3. Conceptual Rationale for EV-Based Therapy over Parent MSCs

The transition from MSC-based to EV-based therapy represents a conceptual shift in regenerative biology. Whereas MSCs require survival, engraftment, and functional integration to exert long-term effects, MSC-EVs act as transient yet potent conveyors of biological information. Delivered locally or systemically, EVs rapidly interact with cardiomyocytes, endothelial cells, and immune cells, delivering functional cargo without the need for cellular persistence [3,6,21].

The cell-free nature of EVs confers several advantages. EVs eliminate risks associated with uncontrolled cell proliferation, ectopic differentiation, and microvascular obstruction, while allowing more predictable dosing and pharmacokinetics [6,7,8]. Moreover, EVs are amenable to standardized manufacturing and quality control, essential prerequisites for clinical translation. Although direct head-to-head comparisons between MSCs and MSC-EVs in identical I/R models remain limited, available data suggest that EVs achieve reductions in infarct size and improvements in cardiac function comparable to those observed with parent cells following single administrations [13,14,17].

## 4. MicroRNA Cargo as a Central Regulatory Layer

A defining feature of MSC-EVs is their enrichment in microRNAs (miRNAs), which act as potent post-transcriptional regulators of gene expression. In myocardial I/R injury, EV-associated miRNAs modulate cardiomyocyte survival, inflammation, oxidative stress, autophagy, and angiogenesis, often targeting multiple pathways simultaneously [22,23,24].

### 4.1. Anti-Apoptotic and Anti-Inflammatory miRNAs

**Figure 1:**
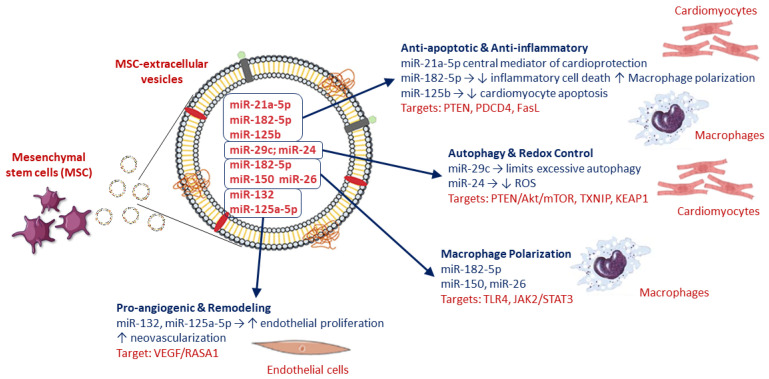
Schematic representation of microRNA present in MSC-EV and their cardioprotective effects. FasL: Fas ligand; PTEN: Phosphatase and Tensin homolog; PDCDP4: Programmed Cell Death Protein 4; mTOR: Mammalian Target of Rapamycin; JAK: Janus kinase; RASA1: RAS p21 protein activator 1; STAT3: Signal Transducer and Activator of Transcription 3; KEAP1: Kelch-like ECH-associated protein 1; VEGF: Vascular Endothelial Growth Factor.

Several miRNAs delivered by MSC-EVs directly suppress apoptotic and inflammatory pathways activated during reperfusion. Exosomal miR-182-5p inhibits inflammatory cell death and promotes macrophage polarization toward reparative phenotypes, thereby limiting inflammatory amplification within the injured myocardium [25,26]. Similarly, miR-125b reduces cardiomyocyte apoptosis and stress signaling by targeting pro-death regulators [27]. Among the most consistently implicated miRNAs, miR-21a-5p emerges as a central mediator of MSC-EV cardioprotection. By converging on core pro-apoptotic and inflammatory signaling nodes, miR-21a-5p enhances cardiomyocyte resilience and functional recovery following I/R injury (Figure 1) [23,27,28].

### 4.2. Autophagy, Oxidative Stress, and Redox Homeostasis

MSC-EV miRNAs also fine-tune autophagic responses during reperfusion. Exosomal miR-29c limits excessive autophagy, preventing maladaptive cardiomyocyte loss [29]. Complementary antioxidant effects are mediated by miR-24 and related miRNAs, which reduce reactive oxygen species (ROS) accumulation and dampen inflammatory signaling (Figure 1) [26,29,30].

### 4.3. Pro-Angiogenic miRNAs and Remodeling Control

Restoration of microvascular integrity is essential for long-term recovery. MSC-EV miRNAs such as miR-132 and miR-125a-5p promote endothelial proliferation and neovascularization, improving perfusion of the peri-infarct myocardium and limiting adverse remodeling (Figure 1) [31,32].

### 4.4. Integrated miRNA Networks

Collectively, available evidence supports a model in which MSC-EV miRNAs act in a coordinated and redundant manner to dampen cardiomyocyte death pathways, restrain excessive inflammation, restore redox homeostasis, and promote vascular repair. Recurrent miRNA families—including miR-21, miR-29, miR-125, miR-132, miR-150, miR-182, and miR-26—converge on a restricted set of molecular nodes controlling apoptosis (PTEN, PDCD4, FasL), oxidative stress (TXNIP, KEAP1), autophagy (PTEN/Akt/mTOR), macrophage polarization (TLR4, JAK2/STAT3), and angiogenesis (VEGF/RASA1) (Figure 1) [24,25,27,33].

Rather than acting in isolation, MSC-EV miRNAs function as integrated regulatory networks that converge on a limited number of survival, inflammatory, and reparative pathways. This redundancy and convergence likely underlie the robustness of MSC-EV–mediated cardioprotection observed across different experimental conditions.

## 5. Protein and Metabolic Cargo of MSC-EVs

In addition to their rich noncoding RNA content, mesenchymal stem cell-derived extracellular vesicles (MSC-EVs) transport a diverse repertoire of proteins that directly support cardiomyocyte survival during I/R injury. Proteomic analyses have identified hundreds of EV-associated proteins involved in cellular metabolism, redox regulation, stress response, and intracellular trafficking, many of which compensate for I/R-induced depletion or dysfunction of critical myocardial proteins [34]. This protein cargo provides a complementary and potentially synergistic layer of cardioprotection alongside miRNA-mediated gene regulation.

### 5.1. Metabolic and Redox Enzymes Supporting Bioenergetic Recovery

**Figure 2:**
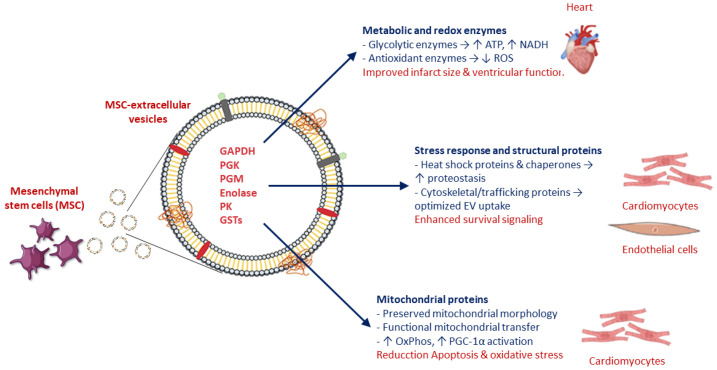
Schematic representation of protein and metabolic cargo of MSC-EV and their cardioprotective effects. GAPDH: glyceraldehyde-3-phosphate dehydrogenase; PGK: phosphoglycerate kinase; PGM: phosphoglucomutase; PK: pyruvate kinase; GSTs: glutathione S-transferases; PGC-1α: Peroxisome proliferator-activated receptor-gamma coactivator 1-alpha.

One of the most consistent findings across proteomic studies is the enrichment of glycolytic enzymes within MSC-derived EVs, including glyceraldehyde-3-phosphate dehydrogenase (GAPDH), phosphoglycerate kinase (PGK), phosphoglucomutase (PGM), enolase, and pyruvate kinase (PK) [34]. In experimental myocardial I/R models, delivery of MSC-EVs restores ATP and NADH levels, indicating functional integration of EV-derived metabolic enzymes into cardiomyocyte energy pathways [10,12]. This metabolic supplementation is particularly relevant during early reperfusion, when mitochondrial oxidative phosphorylation remains impaired and glycolysis becomes a critical determinant of cell survival (Figure 2).

Complementing these metabolic effects, MSC-EVs carry antioxidant enzymes such as peroxiredoxins and glutathione S-transferases (GSTs), which augment endogenous antioxidant defenses and reduce reactive oxygen species accumulation after reperfusion [34]. These redox-modulating proteins align with observed reductions in oxidative stress markers and correlate with smaller infarct size and improved ventricular function in EV-treated hearts.

### 5.2. Stress Response and Vesicle-Associated Structural Proteins

MSC-EVs are also enriched in heat shock proteins and molecular chaperones that enhance cellular tolerance to ischemic and oxidative stress [34]. By facilitating protein folding and preventing aggregation, these chaperones may stabilize cardiomyocyte proteostasis during the abrupt metabolic transition associated with reperfusion.

In addition, vesicle-associated cytoskeletal and membrane-trafficking proteins appear to support efficient EV uptake and intracellular routing, indirectly amplifying downstream survival signaling. Although these proteins are not classical cardioprotective mediators, their presence may optimize cargo delivery and intracellular distribution, thereby enhancing the biological efficacy of MSC-EVs (Figure 2) [34,35].

### 5.3. Insights from Non-MSC EV Protein Cargo: Mechanistic Parallels

Mechanistic support for protein-mediated cardioprotection via EVs also derives from studies using endothelial cell-derived EVs. In a human heart-on-chip ischemia–reperfusion model, endothelial EVs enriched in metabolic, redox, and calcium-handling proteins significantly reduced cardiomyocyte death and preserved contractile function [36]. Although these vesicles were not MSC-derived, the convergence of protein classes reinforces the concept that EV-mediated metabolic and redox supplementation represents a conserved cytoprotective mechanism across EV subtypes.

### 5.4. Mitochondrial Protection and Transfer via EVs: Emerging Evidence

Beyond soluble enzymes, increasing attention has focused on the capacity of EVs to preserve mitochondrial structure and function during I/R injury. In a porcine model of ischemia–reperfusion injury using hearts donated after circulatory death, MSC-EV treatment preserved mitochondrial morphology, reduced cristae disruption, and maintained antioxidant enzyme activity, suggesting protection of mitochondrial integrity despite the absence of direct mitochondrial tracking [37].

More direct evidence for EV-mediated mitochondrial transfer has emerged from closely related cardiac injury models [38,39,40]. Large MSC-derived EVs enriched in mitochondria have been shown to transfer functional mitochondria to injured cardiomyocytes, restoring ATP production, enhancing mitochondrial biogenesis through Peroxisome proliferator-activated receptor-gamma coactivator 1-alpha (PGC-1α) activation, and reducing oxidative stress and apoptosis in vitro (Figure 2) [41]. Similarly, fusogenic plasma membrane vesicles derived from bone marrow MSCs deliver intact mitochondrial components to hypoxia-reoxygenation-injured cardiomyocyte-like cells, improving oxidative phosphorylation and cell survival [41].

Although most direct demonstrations of mitochondrial transfer have been obtained in non-I/R or extra-cardiac models, converging evidence from renal, pulmonary, and cardiac injury studies supports mitochondrial transfer as a biologically plausible and potentially central mechanism of MSC-EV cardioprotection (Figure 2) [39,42,43,44].

### 5.5. Lipid and Metabolite Cargo: Emerging and Understudied Mechanisms

In addition to miRNAs and protein cargo, MSC-derived extracellular vesicles contain bioactive lipids and small metabolites that may contribute to cardioprotective signaling [45]. Lipidomic analyses have shown selective enrichment of sphingolipids, phospholipids, and cholesterol derivatives within EV membranes, suggesting roles in membrane dynamics, vesicle stability, and fusion with recipient cells [46]. Beyond structural functions, specific lipid species may participate in inflammatory modulation and stress signaling pathways relevant to myocardial reperfusion injury [47]. Moreover, EV-associated metabolic intermediates and redox-active molecules may influence cellular bioenergetics and oxidative balance in recipient cardiomyocytes, potentially providing rapid metabolic support during the early phases of reperfusion [48]. While these mechanisms are biologically plausible and supported by broader EV literature, direct evidence linking distinct EV-associated lipids or metabolites to functional cardioprotection in in vivo myocardial I/R models remains limited.

Systematic lipidomic and metabolomic profiling of MSC-EVs in cardiac injury settings will therefore be necessary to clarify the mechanistic and translational relevance of these components.

### 5.6. Integrated View and Remaining Gaps

Taken together, available data indicate that MSC-EV cargo contributes to cardioprotection by restoring metabolic capacity, reinforcing antioxidant defenses, stabilizing cellular stress responses, and preserving mitochondrial structure and function. Unlike miRNAs, which exert regulatory effects over hours to days, protein-mediated actions may provide rapid cytoprotective support during the critical early phases of reperfusion.

However, key gaps remain. The identity of dominant “lead” proteins driving cardiomyocyte survival has not been fully defined, and direct visualization of mitochondrial transfer from MSC-EVs to cardiomyocytes in in vivo myocardial I/R models is still lacking. Addressing these gaps will be essential for refining EV-based therapies and optimizing their translational potential.

## 6. Integration of Pro-Survival and Inflammatory Signaling Pathways

**Figure 3:**
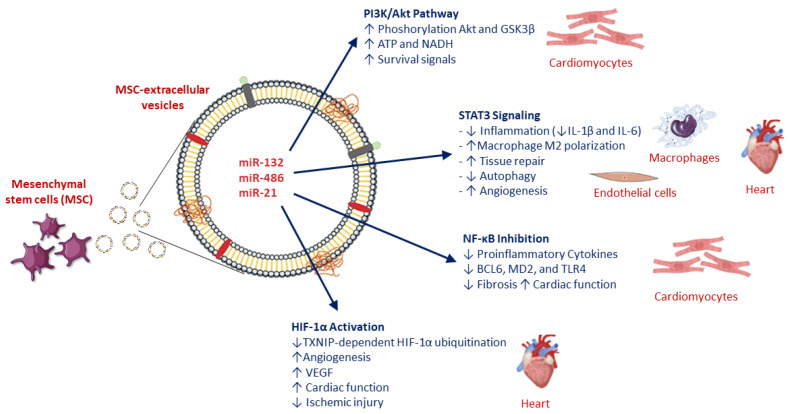
Integrated schematic overview of MSC-EV–mediated cardioprotective mechanisms during myocardial ischemia–reperfusion injury regulated by miRNA. BCL6: B-cell lymphoma 6; MD2: Myeloid Differentiation Protein 2; TLR4: Toll Like Receptor 4; TXNIP: thioredoxin-interacting protein.

MSC-EVs engage a tightly interconnected signaling network that shapes cardiomyocyte fate during reperfusion. Beyond cargo-mediated metabolic and mitochondrial support, MSC-EVs exert cardioprotective effects through the coordinated modulation of canonical pro-survival and inflammatory signaling pathways. Among these, PI3K/Akt, STAT3, NF-κB, and HIF-1α signaling axes have emerged as central and interconnected hubs shaping cardiomyocyte fate, inflammatory responses, and tissue repair during I/R injury (Figure 3) [49].

### 6.1. Activation of the PI3K/Akt Pathway

Robust experimental evidence supports activation of the PI3K/Akt pathway as a core mechanism underlying MSC-EV-mediated cardioprotection in myocardial I/R. In a landmark in vivo study, intravenous administration of MSC-derived exosomes immediately before reperfusion increased myocardial Akt and Glycogen synthase kinase-3 beta (GSK3β) phosphorylation within one hour, concomitant with higher ATP and NADH levels, reduced oxidative stress, a ~45% reduction in infarct size, and improved post-ischemic remodeling. Pharmacological inhibition experiments further confirmed PI3K/Akt signaling as a necessary mediator of these protective effects.

Consistent findings have been reported in cardiomyocyte hypoxia/reoxygenation models, where MSC-EVs activated PI3K and increased the pAkt/Akt ratio, attenuated endoplasmic reticulum stress, and reduced apoptosis. Importantly, blockade of PI3K signaling partially abolished EV-induced protection, directly demonstrating pathway dependence. Additional studies have shown that MSC-EVs activate Akt/mTOR and AMP-activated protein kinase/mammalian target of rapamycin (AMPK/mTOR) signaling to balance autophagy and survival during reperfusion, linking PI3K/Akt activation to both metabolic adaptation and cytoprotection.

Mechanistically, PI3K/Akt activation is largely mediated by EV-delivered noncoding RNAs that suppress PTEN or modulate upstream inflammatory receptors. Multiple MSC-EV miRNAs—including miR-486-5p, miR-132-3p, miR-29c, miR-98-5p, and miR-21—have been shown to converge on PTEN, Tall Like Receptor 4 (TLR4), or Insulin-like Growth Factor-1 (IGF-1) signaling nodes, thereby relieving inhibition of PI3K/Akt and enhancing glucose uptake, ATP production, and resistance to oxidative injury. Collectively, these findings position PI3K/Akt as a central integrator of MSC-EV–induced metabolic and survival signals in the reperfused myocardium (Figure 3) [50,51,52].

### 6.2. STAT3 Signaling: Cell-Specific and Context-Dependent Effects

STAT3 signaling represents a second major axis modulated by MSC-EVs during I/R injury, although its role appears highly cell-type and context dependent. In myocardial I/R models, MSC-EVs enriched in specific miRNAs have been shown to suppress JAK2/STAT3 phosphorylation in cardiac tissue, leading to reduced infarct size, fewer arrhythmias, and lower levels of injury biomarkers. Mechanistic studies revealed that these effects are primarily mediated through macrophage reprogramming, with EV treatment inhibiting M1-like polarization while promoting an anti-inflammatory M2 phenotype, thereby attenuating IL-1β and IL-6 production (Figure 3) [53].

Cross-organ evidence further supports this mechanism. In cerebral I/R models, MSC-EVs reduced infarct volume and improved functional recovery while downregulating JAK2/STAT3 and NF-κB signaling in an AMPK-dependent manner, suggesting a conserved anti-inflammatory signaling module across organs subjected to sterile ischemic injury [54]. In contrast, STAT3 activation by EVs may exert pro-repair effects in non-immune cell populations. In endothelial cells, small EVs derived from induced pluripotent stem cell–derived MSCs activated STAT3 signaling, suppressed excessive autophagy, and promoted angiogenesis; pharmacological inhibition of STAT3 abrogated these benefits [55]. These findings underscore a dual role for STAT3 in MSC-EV biology: suppression of inflammatory STAT3 signaling in macrophages and activation of reparative STAT3 pathways in endothelial cells, jointly contributing to tissue protection and regeneration [56]. HIF-1α signaling emerges as a convergence point linking metabolic adaptation and angiogenesis, particularly in EVs derived from hypoxia-preconditioned MSCs (Figure 3) [57].

### 6.3. NF-κB Modulation and Inflammatory Control

Suppression of NF-κB-driven sterile inflammation constitutes another critical pillar of MSC-EV-mediated cardioprotection. In experimental myocardial infarction and I/R models, MSC-EVs delivering specific miRNAs have been shown to inhibit NF-κB activation through upstream targeting of B-cell lymphoma 6 (BCL6) Myeloid Differentiation Protein 2 (MD2), and TLR4 signaling nodes (Figure 3) [58,59]. This inhibition translated into reduced inflammatory cytokine release, attenuated inflammasome activation, decreased fibrosis, and improved cardiac function

Although some mechanistic insights derive from endothelial or non-cardiac EV sources, a recurring theme emerges across cardiac, cerebral, and hepatic I/R models: EV-mediated disruption of TLR4/MD2-NF-κB signaling dampens innate immune activation and limits secondary tissue damage. In several models, AMPK activation appears to act upstream of NF-κB suppression, linking metabolic sensing to inflammatory control during reperfusion.

Taken together, these findings support NF-κB inhibition as a conserved and biologically relevant mechanism through which MSC-EVs mitigate post-ischemic inflammatory injury and adverse remodeling.

### 6.4. Engagement of HIF-1α Signaling in Ischemic and Reperfused Myocardium

HIF-1α signaling has emerged as a central downstream hub through which MSC-EVs enhance hypoxia tolerance, angiogenesis, and endogenous repair. EVs derived from hypoxia-preconditioned MSCs exhibit superior cardioprotective effects compared with normoxic EVs, largely through miRNA-mediated suppression of TXNIP. By inhibiting TXNIP-dependent HIF-1α ubiquitination and nuclear export, these EVs preserve HIF-1α stability and transcriptional activity under ischemic oxidative stress, reducing cardiomyocyte apoptosis and infarct size (Figure 3) [60].

In parallel, MSC-EVs promote HIF-1α-dependent angiogenesis via delivery of miRNAs targeting factor-inhibiting HIF-1 (FIH1), thereby enhancing HIF-1α nuclear translocation, co-activator binding, and VEGF-driven neovascularization. These effects translate into improved ventricular function, increased capillary density, and reduced ischemic injury in vivo. Additional evidence suggests that MSC-EVs stabilize HIF-1α in resident cardiac stem cells through PTEN/Akt-dependent pathways, supporting stem cell survival and proliferative capacity during ischemia–reperfusion stress (Figure 3).

Collectively, these data position HIF-1α as a key convergence point for MSC-EV-mediated metabolic adaptation, angiogenesis, and cellular survival in the ischemic heart

## 7. Immunomodulation as a Central Axis of MSC-EV Cardioprotection

Rather than acting through isolated pathways, MSC-EVs orchestrate a tightly interconnected signaling network in which PI3K/Akt, STAT3, NF-κB, and HIF-1α pathways dynamically interact. PI3K/Akt activation promotes metabolic recovery and stabilizes HIF-1α; AMPK signaling links energy sensing to suppression of inflammatory JAK2/STAT3 and NF-κB cascades; and context-dependent STAT3 modulation balances inflammation and angiogenesis. This multi-layered signaling integration likely underlies the robustness and reproducibility of MSC-EV-mediated cardioprotection observed across diverse I/R models (Figure 3).

Immunomodulation represents a unifying mechanism of MSC-EV action. MSC-EVs blunt the early inflammatory surge following reperfusion, suppress inflammasome activation, and reduce myocardial levels of IL-1β and IL-18 [25,26]. Macrophages are key mediators of this response, with EV-delivered miR-182 promoting polarization toward reparative M2 phenotypes [25,26].

Neutrophils have emerged as additional targets of MSC-EVs. EVs reduce neutrophil recruitment and suppress neutrophil extracellular trap formation, thereby limiting microvascular obstruction and secondary tissue injury [25]. Together, these findings position immunomodulation as a core component of MSC-EV-mediated cardioprotection.

## 8. Clinical Translation and Emerging Applications

Although most mechanistic evidence derives from preclinical models, early translational investigations of MSC-derived extracellular vesicles in cardiovascular settings have begun to delineate their clinical feasibility and biological activity. In regenerative cardiology, small exploratory studies and proof-of-concept approaches have demonstrated acceptable safety profiles and signals of biological engagement, including modulation of inflammatory markers, improvement in surrogate measures of ventricular function, and attenuation of adverse remodeling indices [61,62]. However, clinical outcomes have shown variability, reflecting heterogeneity in EV isolation protocols, dosing strategies, timing of administration, and patient selection.

In the context of acute myocardial infarction, EV-based approaches have been explored as adjunctive therapies aimed at reducing infarct size and limiting reperfusion-associated damage [63]. While some translational models and early human investigations suggest potential benefits in terms of myocardial salvage and inflammatory modulation, results remain inconsistent and insufficient to support routine clinical implementation [64]. Furthermore, challenges related to large-scale production, batch-to-batch variability, route of delivery (intracoronary versus intravenous), and optimal therapeutic window remain unresolved. The development of engineered EVs enriched with defined cardioprotective cargoes represents an emerging strategy to enhance biological potency and reproducibility [19].

Overall, current evidence suggests that MSC-derived EVs are biologically active and clinically feasible, yet definitive demonstration of robust and reproducible cardioprotective efficacy in myocardial ischemia–reperfusion injury is still pending. Carefully designed, adequately powered clinical trials will be required to determine whether the promising mechanistic profile observed in experimental models can translate into meaningful clinical benefit.

## 9. Future Perspectives

The field of MSC-derived extracellular vesicles in myocardial ischemia–reperfusion injury has reached several areas of emerging consensus, while important biological and methodological uncertainties remain. Experimental evidence consistently supports the predominance of paracrine mechanisms, the integration of pro-survival and anti-inflammatory signaling pathways, and the contribution of mitochondrial and metabolic support during early reperfusion. There is also growing agreement that EV-based strategies may overcome several limitations inherent to cell-based therapies. However, significant challenges continue to limit translational progression. Variability in EV isolation and purification methods results in heterogeneous vesicle populations with differing cargo composition and biological activity [65,66,67,68].

The absence of standardized potency assays complicates cross-study comparisons and limits reproducibility. In addition, most available data derive from small rodent models, which may not fully recapitulate the metabolic, immunological, and electrophysiological complexity of human myocardial ischemia–reperfusion injury.

Addressing these biological and methodological gaps will be essential to translate experimental findings into clinically meaningful therapies.

The accumulated experimental evidence reviewed herein supports MSC-derived EVs as multifunctional biological mediators capable of mitigating myocardial I/R injury through coordinated modulation of survival, metabolic, inflammatory, and reparative pathways. Importantly, MSC-EVs do not act through a single dominant mechanism; rather, they reprogram the injured myocardium at multiple levels, influencing cardiomyocytes, endothelial cells, and immune populations in a temporally coordinated manner.

From a conceptual standpoint, MSC-EVs exemplify a shift from cell replacement toward information-based therapy. Their cargo integrates microRNA-mediated gene regulation, protein-driven metabolic support, mitochondrial protection, and immune rebalancing. This integration likely explains the robustness and reproducibility of MSC-EV-mediated cardioprotection across diverse experimental models. Nevertheless, several biological and translational challenges remain. EV heterogeneity, influenced by MSC source, donor variability, and conditioning protocols, complicates standardization.

The efficacy of MSC-EVs depends on timing, dose, and delivery route. Experimental evidence suggests that administration during early reperfusion maximizes biological impact [69]. To extend therapeutic exposure, injectable hydrogels and epicardial patches have been developed to enable sustained EVs release, improving myocardial retention and limiting adverse remodeling [70].

The relative contribution of individual cargo components, miRNAs vs. proteins vs. metabolic factors, remains incompletely resolved and may vary across injury contexts. Moreover, the optimal therapeutic window, dosing strategies, and long-term safety profiles of MSC-EVs require further systematic investigation.

Despite these challenges, the advantages of EV-based therapy (cell-free delivery, reduced immunogenicity, scalable manufacturing, and compatibility with off-the-shelf use) position MSC-EVs as promising candidates for next-generation cardioprotective interventions. Future studies integrating high-resolution EV profiling, cell-specific functional assays, and large-animal models will be essential to translate these biological insights into clinically effective therapies.

## 10. Conclusions

The experimental evidence reviewed in this work supports mesenchymal stem cell–derived extracellular vesicles as central mediators of cardioprotection in myocardial ischemia–reperfusion injury. Rather than acting through a single dominant pathway, these vesicles exert pleiotropic effects by coordinating multiple interconnected biological mechanisms that govern cardiomyocyte survival, metabolism, inflammation, and tissue repair. Through the delivery of regulatory microRNAs, proteins, and metabolic enzymes, extracellular vesicles modulate key signaling axes such as phosphoinositide 3-kinase and protein kinase B, signal transducer and activator of transcription 3, nuclear factor kappa B, and hypoxia-inducible factor 1, while preserving mitochondrial structure, energy production, and quality control processes, including mitophagy.

This integrated mode of action distinguishes extracellular vesicle–based approaches from conventional pharmacological strategies that target isolated molecular pathways and may explain the robustness and reproducibility of their protective effects across diverse experimental models of ischemia–reperfusion injury. Importantly, extracellular vesicles overcome several limitations associated with cell-based therapies, including issues related to cell survival, engraftment, and safety, while offering greater opportunities for standardization and controlled delivery.

Despite these advantages, significant challenges remain. Extracellular vesicle heterogeneity, influenced by cell source, conditioning protocols, and cargo composition, complicates mechanistic prioritization and translational development. Further studies integrating high-resolution vesicle profiling, cell-specific functional analyses, and large-animal models are required to define optimal dosing, timing, and delivery strategies. Addressing these issues will be essential to advance extracellular vesicle–based therapies toward clinically viable, mechanism-driven interventions for myocardial ischemia–reperfusion injury.

## Data Availability

No new data were created or analyzed in this study. Data sharing is not applicable.
